# Cervical Spondylotic Myelopathy Presenting as Ischemic Stroke: A Case Report

**DOI:** 10.7759/cureus.4291

**Published:** 2019-03-21

**Authors:** Ogenetega J Madedor, Scott Lee, Robert Levey

**Affiliations:** 1 Internal Medicine, The Brooklyn Hospital Center, Academic Affiliate of the Icahn School of Medicine, Clinical Affiliate of the Mount Sinai Hospital, Brooklyn, USA

**Keywords:** cervical myelopathy, stroke, degenerative, spinal stenosis

## Abstract

Cervical spondylotic myelopathy (CSM) is a condition seen in individuals greater than 50 years of age and is often asymptomatic. In patients who are symptomatic, the symptoms include cases where patients may present with paresis, neck stiffness, and gait abnormalities. We present a 63-year-old male who complained of a four-week-long "tingling and numbness" in his right upper and lower extremities. The sensation worsened over the next couple of days to the point where it affected his gait and led to a subsequent visit to the emergency room. Initial presentation prompted a stroke workup, but further investigation revealed findings suggestive of CSM. This case report highlights the symptomology of cervical spondylotic myelopathy in adults greater than 50 years of age and emphasizes the importance of recognizing the essential markers of this condition.

## Introduction

Cervical spondylotic myelopathy (CSM) is the result of degenerative processes within the cervical spine that lead to cord compression [1]. Risk factors include age greater than 50 years, history of cervical neck trauma, and degenerative bone diseases [1]. Common symptoms of CSM are balance abnormalities, arm weakness, numbness of the hands, leg stiffness, urinary urgency, and neck pain [2]. Disease progression varies due to interpersonal factors along with environmental factors, which can influence bone degeneration. The prevalence of CSM is higher in men than in women [3]. It is estimated that the prevalence of degenerative cervical myelopathy is 605 per million and the incidence is 41 per million in North America [4]. The incidence of CSM-related hospitalizations was estimated to be 4.04 per 100,000 person-years [5]. A study conducted in Asia identified a genetic link between CSM and factors of the vitamin D receptor, collagen IX, and enzyme MMP-2 [4]. Herein, we report a case of CSM presenting as ischemic stroke.

## Case presentation

A 63-year-old Albanian man with a past medical history of prostate cancer status post prostatectomy presented with tingling and numbness in his right upper and lower extremities. These symptoms were found to have persisted for at least a month. He denied smoking, alcohol use, and drug use. The patient’s daughter noticed he had an unsteady gait and required assistance when moving, which prompted an immediate visit to the emergency room.

**Figure 1:**
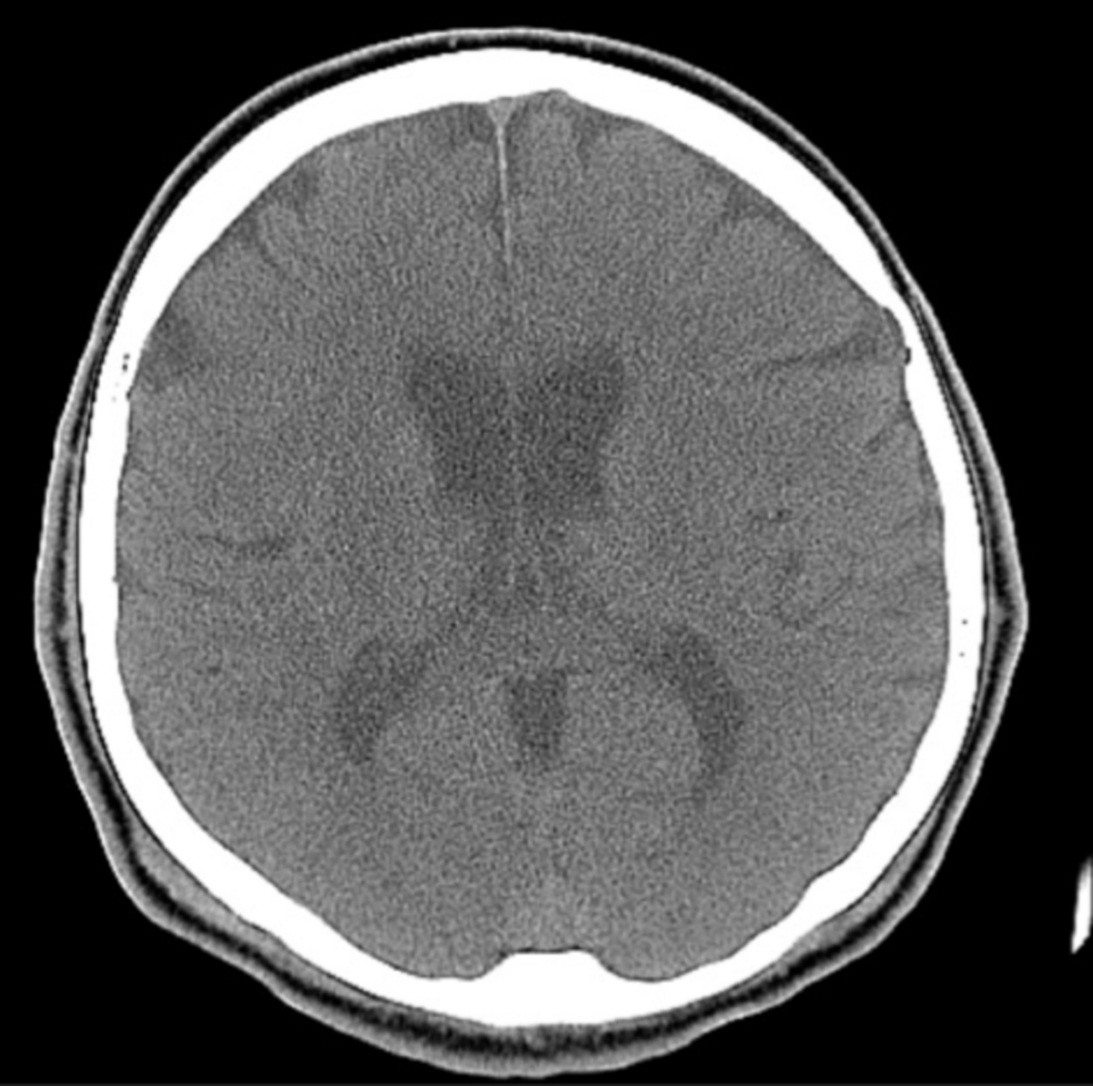
CT of the head No acute ischemic or hemorrhagic infarcts, midline shift, hydrocephalus, herniation, or old infarcts. CT: computed tomography

**Figure 2:**
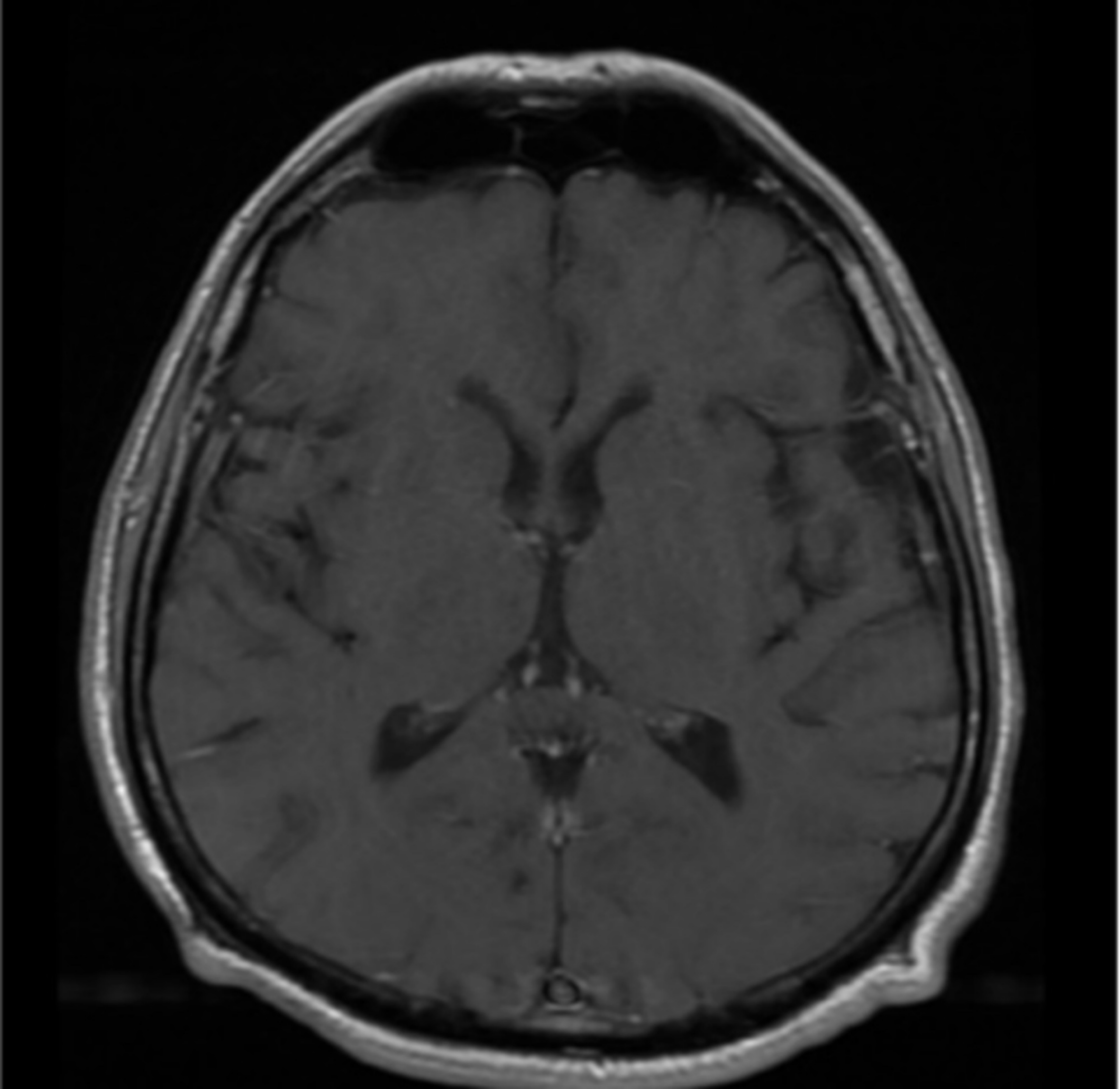
Axial T2 FLAIR image of the brain without contrast No evidence of an acute infarction or abnormal intraparenchymal enhancements or masses. The craniocervical junction (not pictured) was within normal limits. FLAIR: fluid attenuated inversion recovery

On the day of admission, his vital signs were a temperature of 97.9 F, heart rate of 68 beats/min, blood pressure of 137/84 mmHg, and respiratory rate of 18 breaths per minute. Upon arrival, the patient was both alert and oriented to person, place, and time. On neurological examination, he showed right-sided hemiparesis with significant leg stiffness. The patient’s strength in his right arm was 4/5 but the strength in his right leg was 3/5. The left upper and lower extremities were unremarkable. Cranial nerves 2-10 showed no deficit, however, there was difficulty with shrugging the right shoulder. He was also unable to turn his head in the left direction. The patient showed an unsteady gait upon walking, which was compensated by his non-affected side. The rest of the physical examination was within normal limits. Laboratory values were within normal limits. The initial head computed tomography (CT) and follow-up magnetic resonance imaging (MRI) were negative for a stroke (Figures 1-2).

**Figure 3:**
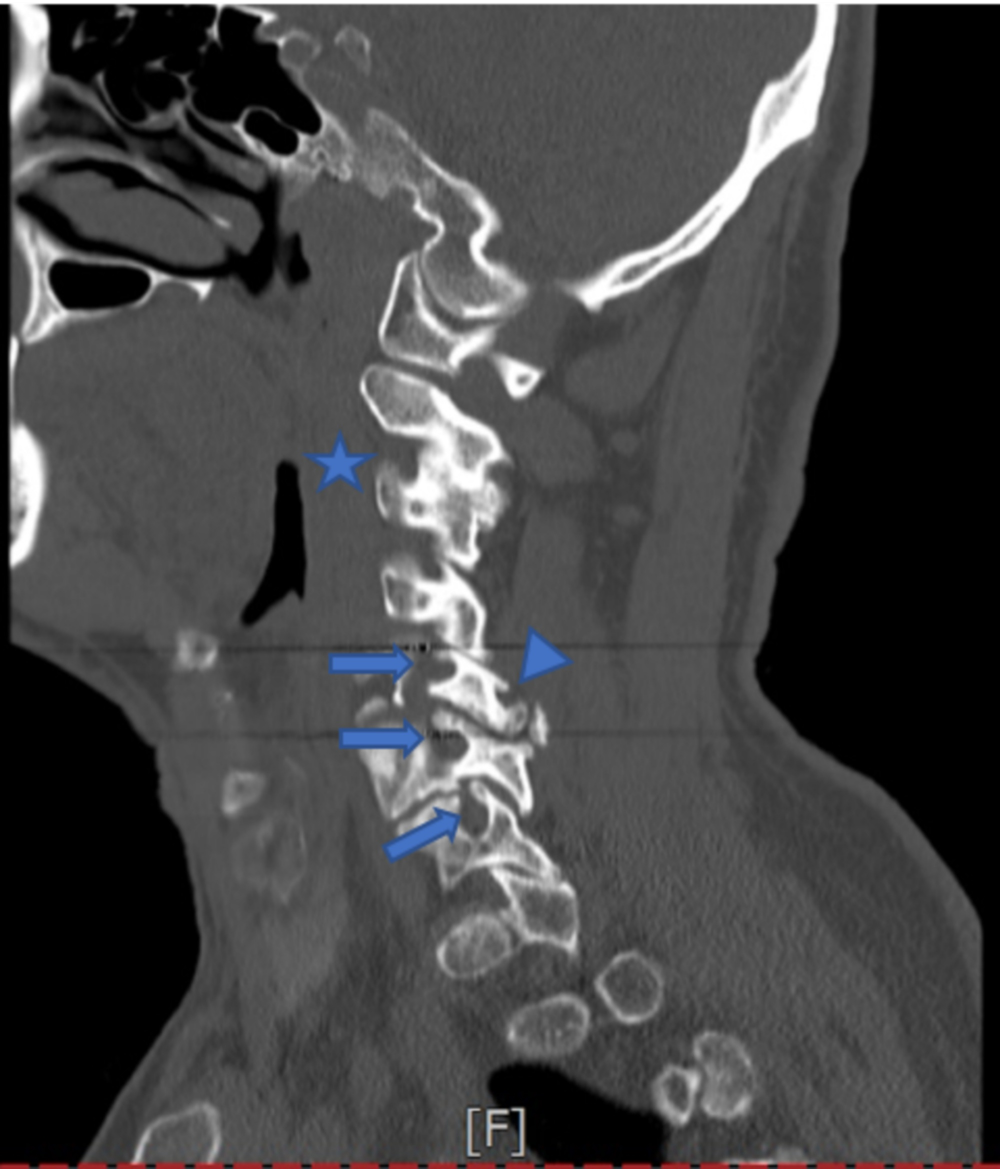
CT of the cervical spine Left posterior fusion of C2-C3 (star). Moderate to severe canal stenosis of C4-C5 and C5-C6 (arrows). At C5-C6, there is grade 1 anterolisthesis with uncovering of the disc and broad-based disc osteophyte complex (triangle). At C6-C7, there is broad-based osteophyte complex and bilateral uncovertebral. CT: computed tomography

**Figure 4:**
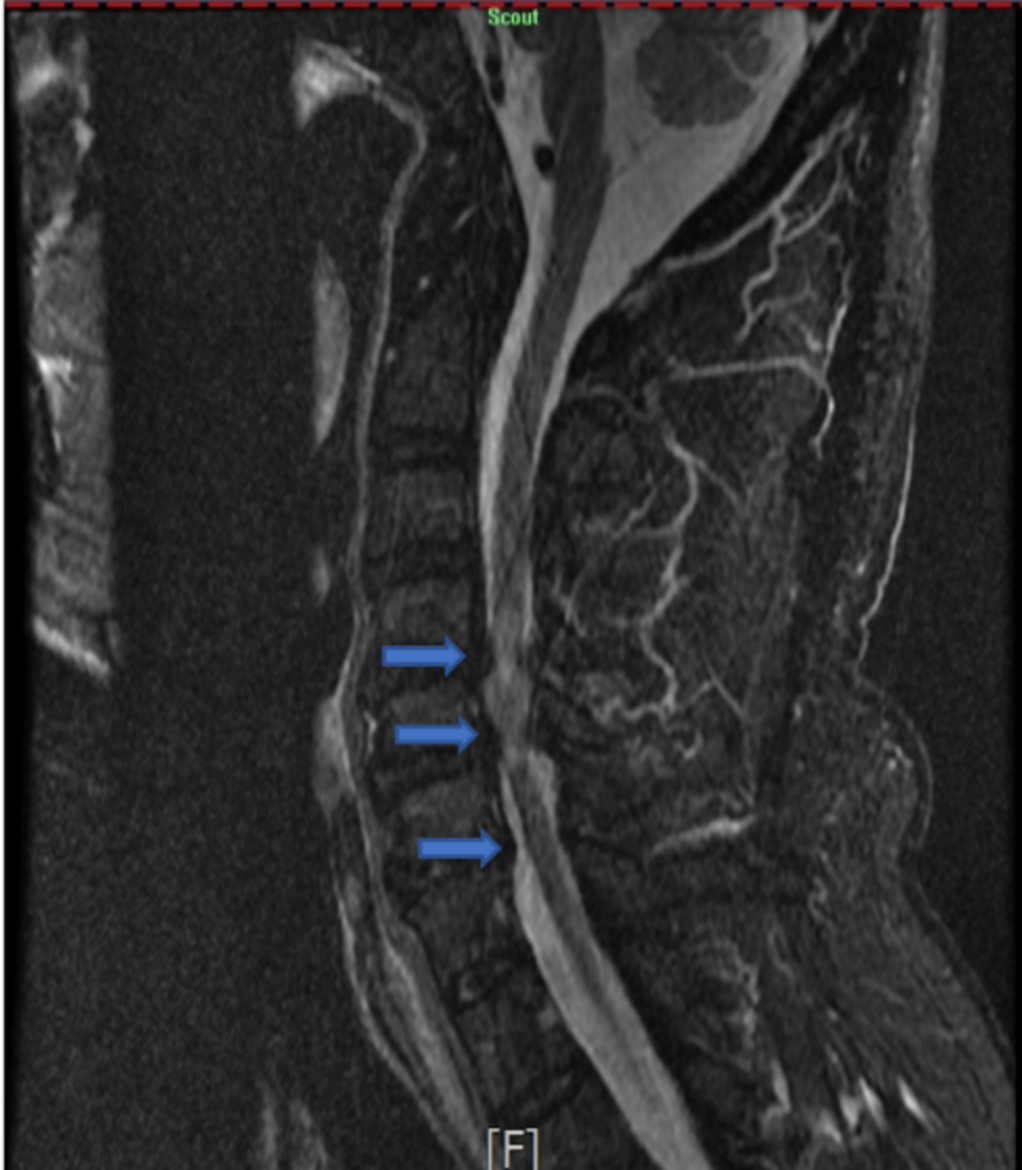
STIR image of the cervical spine Severe spinal stenosis at C4/C5 and C5/C6 causing increased cord signal and cord compression. STIR: short TI inversion recovery

CT of the cervical spine showed marked degenerative changes, including moderate to severe spinal canal stenosis at C4/C5 (Figure 3). Subsequent MRI of the cervical spine was performed without contrast, which showed moderate to severe central stenosis with cord compression and mildly increased T2 signal at C4/C5 and C5/C6 (Figure 4).

There were degenerative findings between levels C3-C7 along with hypertrophy of the facet joints. He was started on 1000 mg solumedrol. Shortly after, the patient’s range of motion improved in the upper extremities. He also stated that he was no longer feeling weakness in the right hand. However, leg stiffness and unstable gait persisted. He was recommended fusion of C4/C5 and C5/C6 vertebra for long-term treatment and increased quality of life.

## Discussion

CSM is a treatable condition if detected early in its disease course. However, clinicians may have up to a two-to-five-year delay of diagnosis depending on the presenting symptomology [6]. In our case report, the patient presented with stroke symptoms and sudden balance impairment, thus prompting the stroke assessment protocol. Further neurologic workup and imaging lead to the ruling out of the initial differential and diagnosing the patient with CSM.

The initial progression of the CSM causes subtle gait disturbances, which, on physical examination, can be confused for spastic movements [7]. In addition, patients report minor sensory deficits in the hand and wrist [1,7]. Patients usually describe difficulty with writing, holding objects, and diffuse numbness in the hand. These findings usually don’t prompt an individual to visit their primary care physician because they do not have any associated pain. Minor deviations in gait with hand abnormalities may not be deemed physician-worthy from the individual’s perspective. Often, patients are misdiagnosed with carpal tunnel, fibromyalgia, or unspecific peripheral neuropathy, continuing to delay necessary care [2,6].

In a retrospective study of 42 patients with CSM, patients had a greater than two-year delay until they were diagnosed, even though they saw their family physician or orthopedic surgeon more than five times within that time span [8]. When the patients in this study saw their family physician, they would often be given symptomatic care with non-steroid anti-inflammatory drugs, steroids, and other neuropathic drugs. These would initially decrease the likelihood of exacerbations but did not fix or delay progression of the disease. Thus, these patients would follow up with an orthopedic surgeon and/or neurologist when their symptoms returned and continue this cycle until someone eventually put the pieces of the puzzle together.

Physical examination is essential for arriving at the correct diagnosis of CSM. Patients shown to have sensory abnormalities in the hands should be assessed by the grip and release test. Individuals with myelopathy will have difficulty with the repetitive task due to weakness and hand dexterity. Severe deficits in hand dexterity can be measured with the nine-hole peg test, which correlates with both the progression of upper extremity function and the improvement of symptoms with treatment [9]. The Lhermitte phenomenon is a test that should be done if someone presents to the clinic with an abnormal gait and peripheral neuropathy. Flexion of the neck causes the stretching of the hyper-excitable demyelinated dorsal column of the spinal cord, resulting in electric-shock that radiates down through the body [10-11]. A previous study found this to be 97% specific for nonspecific compressive myelopathy [12]. The Romberg test is another assessment tool that can be used to differentiate between motor deficit or neurological abnormality for unsteady gait. Testing reflexes allows the physician to elicit hyperreflexia and clonus, which are hallmarks of CSM [1,13-14].

MRI is the best initial test for CSM because it can identify spinal cord inflammation, edema, lesions, and high signal changes [1,14]. An MRI scan can identify disc herniation, which can be seen as a narrowing of the spinal canal in certain segments [1]. CT is more accurate than MRI in evaluating canal stenosis because of the superior imaging of the bone. Compression due to ossification or osteophytes of the posterior longitudinal ligament is best visualized on CT [1,13-14]. Conservative management, usually given according to clinician preference, such as non-inflammatory medication, steroids injections, lifestyle modifications, and neck immobilization collars are indicated [13]. However, once the condition becomes severe and myelopathy is observed in the patient, conservative management limits patient recovery and does not slow progression. Only surgical intervention has been shown to slow the progression of spinal degeneration, decrease acute pain flares, and increase the range of motion in the upper extremities [2].

Indications for surgery are cases refractory to conservative treatment, progressive deterioration due to symptoms, the presence of myelopathy for greater than six months, compression ratio around 0.4, which correlates to a flattened spinal cord, a transverse area of the cord less than 40 mm, and bladder or bowel dysfunction [13-14]. The main surgical goal would be cervical decompression, done either by the anterior, posterior, or combined technique [2,14]. Both the anterior and posterior approach can address kyphosis due to compression, however, only the anterior can correct indirect compression due to osteophyte formation [1-2]. This makes the anterior method the preferred method [1,14]. The posterior approach is indicated for multisegment stenosis and dorsal compression due to the infoldings of the ligamentum flavum [14]. If left untreated, complications of CSM include severe sensory deficits, wheelchair dependence, and quadriplegia [2].

## Conclusions

CSM is a known cause of impairment in the elderly population. Disease progression can be linear, variable, or even static, which makes correlating symptoms with a diagnosis challenging. Several differentials can be applicable, which means clinical professionals need to be able to siphon through past medical history and clinical manifestations. Furthermore, clinicians should be aware that CSM can present as ischemic stroke as well as other neurologic disorders.
